# Causality between coronavirus disease 2019 and tuberculosis: A two-sample Mendelian randomization study

**DOI:** 10.1097/MD.0000000000041999

**Published:** 2025-04-25

**Authors:** Lei Wang, Chidao Chen, Peng Wang, Fan Wang, Xixin Wang, Fengzhu Sun, Yanru Ji, Zhonghao Huang

**Affiliations:** a College of Medical Imaging, Qilu Medical University, Zibo, Shandong, China.

**Keywords:** COVID-19, Mendelian randomization, tuberculosis

## Abstract

An association between coronavirus disease 2019 (COVID-19) and tuberculosis (TB) has been reported, although the causal relationship between these factors remains unclear. This study aimed to investigate the causal connection between COVID-19 and TB using Mendelian randomization (MR) analysis. The UK Biobank provided summary data on COVID-19 using the integrative epidemiology unit open genome-wide association studies (GWAS) pool. GWAS data on TB were also retrieved. The relationship between COVID-19 and TB was examined using 5 methods, the major method being inverse variance weighting. Additional methods included weighted median, MR-Egger regression, simple mode, weighted mode, and Wald ratio. No significant correlation was observed between COVID-19 and TB (odds ratio = 1.022, 95% confidence interval 0.955–1.032, *P* = .611). Reverse MR analysis also confirmed the absence of a causal relationship between COVID-19 and TB risk (*P* > .05). This study used several complementary MR approaches to explore the bidirectional relationship between COVID-19 and TB and revealed no significant bidirectional relationship. However, given the limited GWAS data for these 2 conditions, caution is warranted when interpreting these results. While previous epidemiological and retrospective studies have suggested that COVID-19 may impact TB, our bidirectional MR analysis based on European population genetic data suggests no two-way causality between COVID-19 and TB in this population.

## 1. Introduction

Since the onset of coronavirus disease 2019 (COVID-19) in late 2019, the pandemic has profoundly impacted the global public health systems.^[1]^ Tuberculosis (TB), one of the deadliest infectious diseases worldwide, presents unprecedented challenges. According to reports from the World Health Organization, TB induced 1.4 million deaths in 2019, making it the main cause of death from a unique infectious disease worldwide.^[2]^ However, the spread of COVID-19 has led to significant disruptions in TB diagnosis and treatment services, with the number of new TB cases dropping to 5.8 million globally in 2020, an 18% decrease from 2019.^[3]^ This situation not only hindered TB prevention and control efforts but also worsened the health conditions of patients with TB.

Recent studies have suggested a link between COVID-19 and TB. Although previous studies have examined how COVID-19 affects TB, they primarily consisted of descriptive statistics and retrospective studies, lacking in-depth investigations into the causal relationships between the 2, and may be affected by reverse causality and complicated confounding variables.^[4–7]^ Systematic studies on the epidemiological characteristics, treatment outcomes, and mutual effects of COVID-19 and TB co-infection are scarce in the current literature. This research gap poses challenges for formulating effective public health policies, particularly in resource-limited settings. Mendelian randomization (MR) is an alternative statistical method that uses single nucleotide polymorphism (SNPs) as instrumental variables (IVs) to explore causal relationships between exposures and outcomes, free from confounding effects.

This study aimed to evaluate the MR analysis technique to examine the causality between COVID-19 and TB, which is crucial for developing effective public health strategies and interventions.

## 2. Materials and methods

### 2.1. Study design

**Figure 1.**
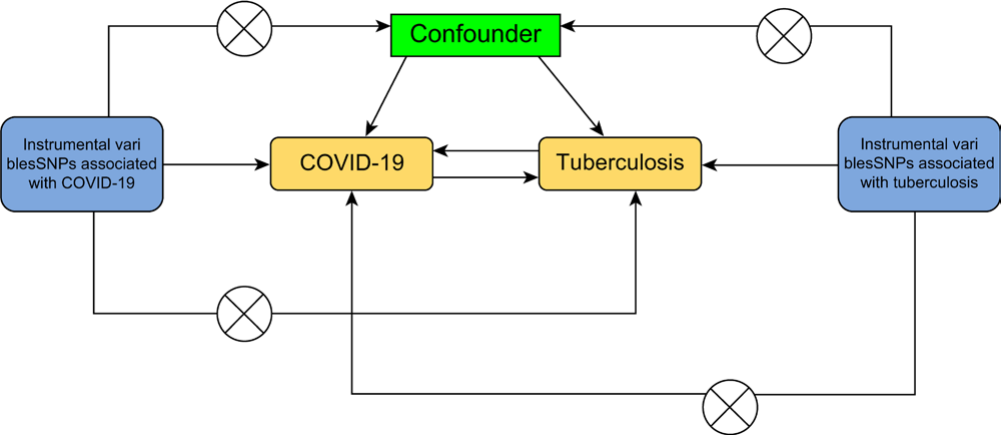
The model of MR analysis. MR = Mendelian randomization.

This study evaluated the causality between COVID-19 and TB using a two-sample MR technique with publicly accessible genome-wide association studies (GWAS) data, with COVID-19 as the exposure and TB as the outcome. Following established guidelines, SNPs significantly associated with COVID-19 were selected as IVs, and heterogeneity and horizontal pleiotropy tests were performed. Finally, to exclude the probability of TB influencing COVID-19, a reverse MR analysis was performed. The analysis satisfied the following 3 assumptions: (1) IVs are strongly associated with exposure, (2) confounding factors do not affect IVs, and (3) IVs influence the outcome only through exposure. The data used in this project were derived from publicly available GWAS datasets. All included study data were approved by the North West Multi-center Research Ethics Committee (REC reference: 21/NW/0157). The need to obtain informed consent was waived due to the retrospective nature of the study (Fig. 1).

### 2.2. Data sources

This study used COVID-19 summary data retrieved from the Integrative Epidemiology Unit Open GWAS database, whereas TB GWAS data were obtained from the UK Biobank, a large biomedical research database that collects clinical and genetic data from over 500,000 UK residents to enhance research on human health and disease. The TB dataset included 2277 European cases and 460,656 controls, whereas the COVID-19 dataset included 6406 European cases and 902,088 controls. The results are unlikely to be skewed because the exposure and outcome data samples have little overlap (Table 1).

**Table 1:** Mendelian randomization genome-wide association study data of the 2 samples.

Project	Samples	SNPs	URL
COVID-19	908,494	12,832,272	https://gwas.mrcieu.ac.uk/
Tuberculosis	462,933	9,851,867	https://www.ukbiobank.ac.uk/

COVID-19 = coronavirus disease 2019, SNP = single nucleotide polymorphism, URL = uniform resource locator.

### 2.3. Selection of IVs

Because of the small number of SNPs obtained using the threshold (*P* < 5 × 10^−8^), the threshold was adjusted to (*P* < 5 × 10^−6^) following previous studies, with a linkage disequilibrium threshold of r2 < 0.001 and a clumping window of 10,000 kb. When palindromic SNPs were present, the minimum allele frequency threshold was set to 0.3. The PhenoScanner database was used to check whether the selected IVs were affected by confounding factors, and the IVs that violated assumptions (2) and (3) were excluded. Reverse MR analysis utilized a more lenient criterion of *P* < 5 × 10^−7^ to identify IVs, owing to the increased number of SNPs.

### 2.4. Analytical process

This study employed 5 MR analytical methods to assess causal relationships: inverse variance weighted (IVW), weighted mode (WM), MR-Egger regression, simple median (SM), and weighted median. The IVW method provides better results when SNPs do not exhibit horizontal pleiotropy by weighing the results of each study according to their variance.^[8]^ MR-Egger regression is used to detect and correct IV bias in MR analysis, which is suitable for cases where IVs exhibit pleiotropy.^[9]^ The SM and WM methods aggregate SNPs with similar causal effects back into clusters.^[10]^ Sensitivity analysis and statistical tests, mainly the MR-Egger intercept, leave-one-out analysis, and Cochran Q test, were performed to measure stability and horizontal pleiotropy. Finally, the SNPs associated with TB were used as IVs for reverse MR analysis to investigate potential reverse causation.

### 2.5. Statistical analysis

The data were examined using the TwoSampleMR program in R. (v 4.3.1). 5 MR analysis methods (MR-Egger, SM, IVW, WM, and weighted median) were employed, with IVW^[11]^ as the primary method and the other 4 as supplementary methods to elucidate the specific relationship between exposure and outcome. Beta values were calculated as odds ratio with 95% confidence interval. Horizontal pleiotropy was assessed using the MR-Egger intercept test. If the intercept term was equal to zero, horizontal pleiotropy was considered absent.^[12]^ Cochran Q test was used to identify the heterogeneity of the SNPs.^[13]^ Sensitivity analyses were conducted using the leave-one-out method to compute the combined effect size and evaluate whether any single IV could drive a causal association (*P* < .05).

## 3. Results

### 3.1. MR analysis results

**Figure 2.**
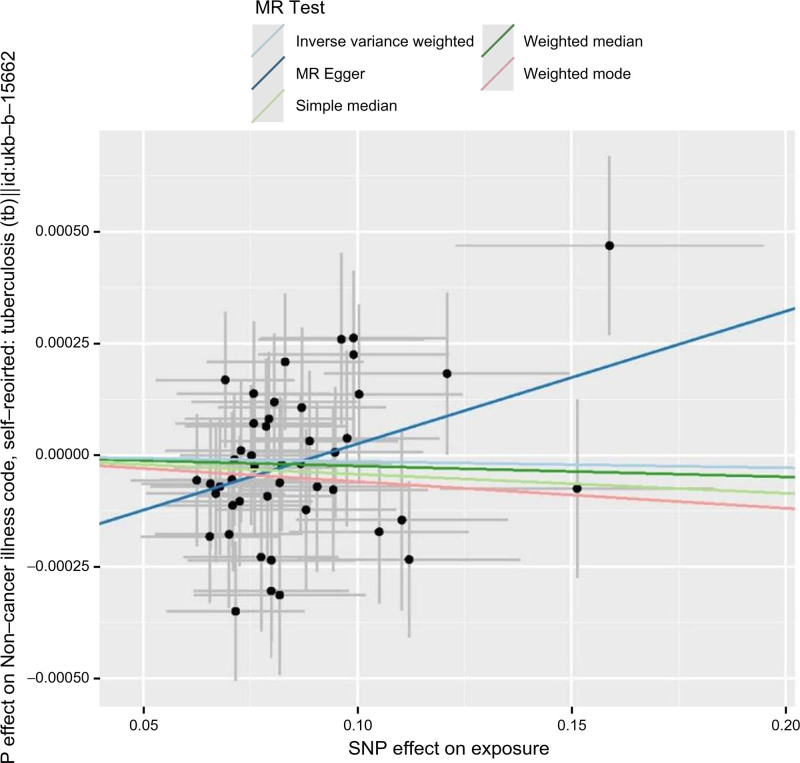
Scatter plot of 5 MR analysis models of COVID-19 and tuberculosis. MR = Mendelian randomization, COVID-19 = coronavirus disease 2019.

Five different MR methods were employed for the two-sample analysis of COVID-19 and TB, indicating no association (odds ratio = 1.022, 95% confidence interval = 0.955–1.032, *P* = .611) (Table 2). 46 SNPs were used as IVs to examine the connection between COVID-19 and TB, with the IVW method yielding *P* > .05. The correlation coefficient between COVID-19 and TB was −0.000142, indicating no association (Fig. 2).

**Table 2:** Results of Mendelian randomization analysis between coronavirus disease 2019 and tuberculosis.

Exposure	Method	SNPs	Beta	OR	95%CI	*P*
	MR Egger	46	0.003	1.003	0.958–1.012	3.29E−02
	Weighted median	46	−0.000244173	1	0.873–1.056	5.42E−01
COVID-19	Inverse variance weighted	46	−0.000142487	1.022	0.955–1.032	6.11E−01
	Weighted mode	46	−0.001	0.999	0.999–1.001	5.47E−01
	Simple median	46	−0.000427706	1	0.926–1.012	2.93E−01

CI = confidence interval, COVID-19 = coronavirus disease 2019, OR = odds ratio, SNP = single nucleotide polymorphism.

### 3.2. Quality control

**Figure 3.**
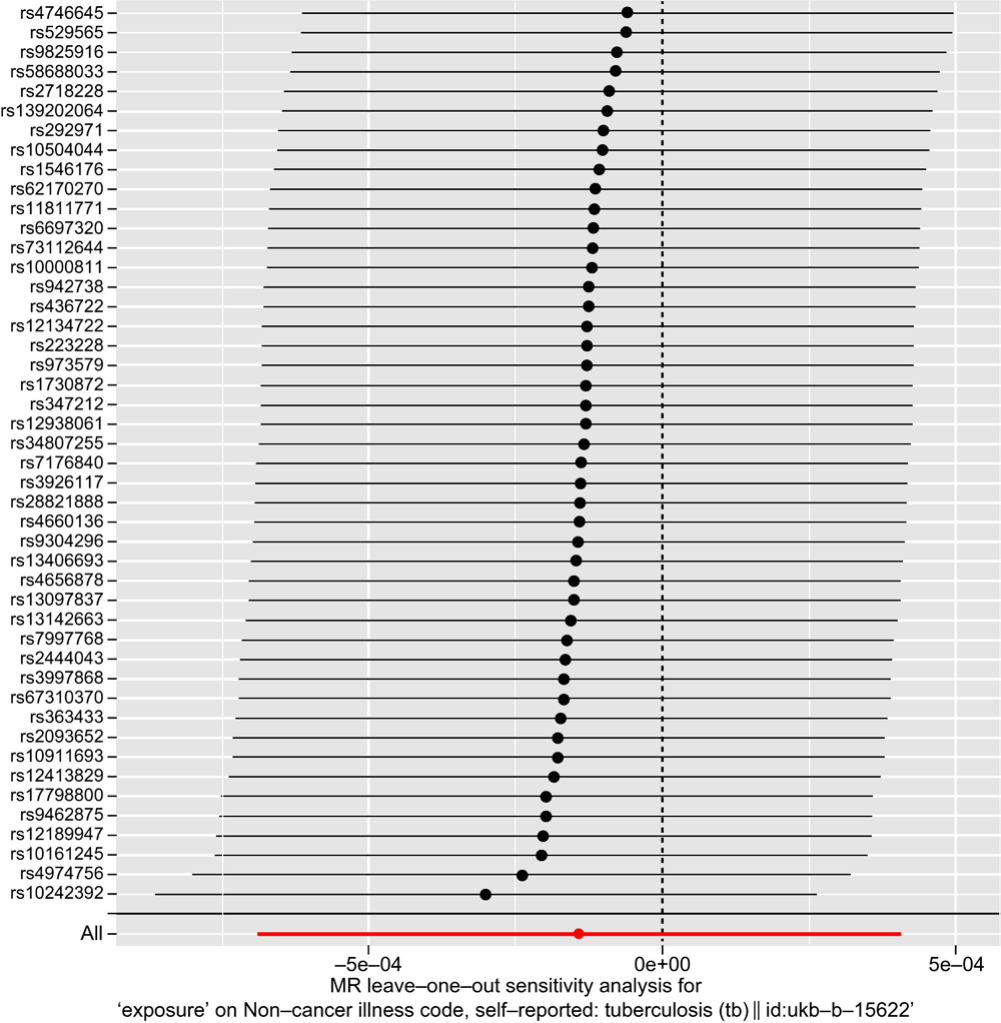
Leave-one-out analysis of the causal relationship between COVID-19 and tuberculosis. COVID-19 = coronavirus disease 2019.

**Figure 4.**
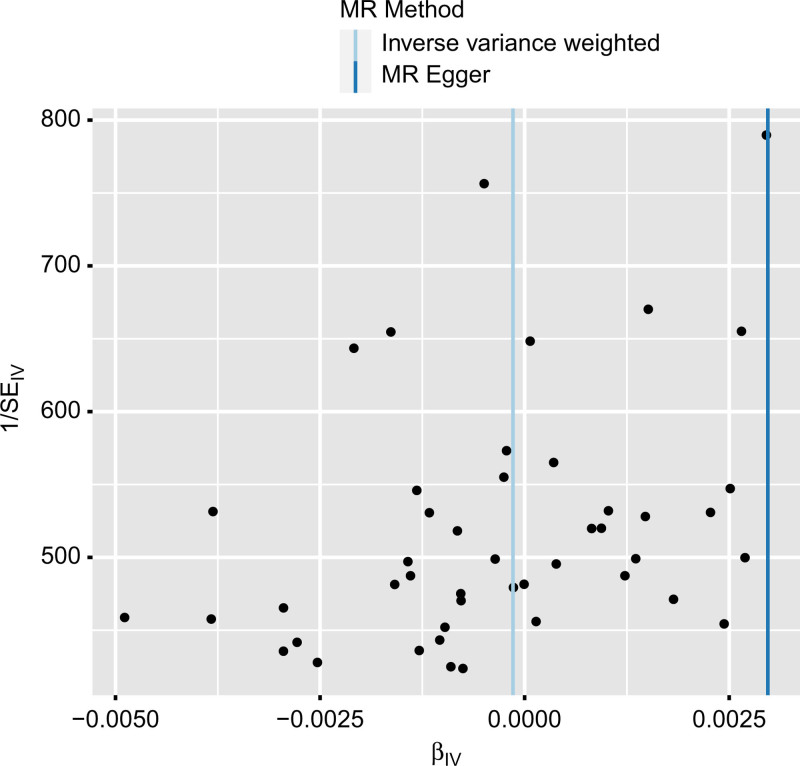
Funnel plot analysis of the causal relationship between COVID-19 and tuberculosis. COVID-19 = coronavirus disease 2019.

We performed tests for horizontal pleiotropy and heterogeneity (*P* > .05) (Table 3). Leave-one-out analysis revealed that the effect sizes of most IVs were very close to the total effect size, confirming that the causal association between COVID-19 and TB was not driven by a single IV, thus ensuring the stability of the MR results; funnel plots were used to assess heterogeneity (Figs. 3 and 4).

**Table 3:** Quality control findings of the link between tuberculosis and coronavirus disease 2019.

Exposure	Horizontal pleiotropy		Heterogeneity test		
	MR-Egger		Cochran Q		
COVID-19	Egger_intercept	*P*	Method	Q	Q_pval
	0.0002393	5.11e−01	MR Egger	19.040	2.47e−01
	_	_	Inverse variance weighted	13.245	3.82e−01

COVID-19 = coronavirus disease 2019, MR = Mendelian randomization.

### 3.3. Reverse MR analysis

Additionally, the reverse MR analysis’s findings showed no meaningful correlation between TB and COVID-19 (*P* > .05) (Table 4).

**Table 4:** Reverse Mendelian randomization analysis.

Exposure	Method	SNPs	Beta	*P*
	MR Egger	3	−0.00211	.623
	Weighted median	NA	NA	NA
Tuberculosis	Inverse variance weighted	3	−0.007159	.687

MR = Mendelian randomization, SNP = single nucleotide polymorphism.

## 4. Discussion

This study used several complementary MR approaches to discover the bidirectional relationship between COVID-19 and TB and revealed the absence of a significant bidirectional relationship. However, given the limited GWAS data for these 2 diseases, caution should be exercised when interpreting the results. Previous epidemiological and retrospective studies have suggested that COVID-19 may impact TB; for instance, Tadolini et al^[14]^ found that COVID-19 could activate latent TB in patients, with a significantly higher early mortality rate in co-infected patients. In a retrospective study involving 223 COVID-19 patients, 34 of whom had TB, Wei et al^[15]^ found that those with severe or critical COVID-19 had a higher mortality rate and more severe conditions than those without TB. Wang et al^[16]^ conducted a systematic review and meta-analysis that indicated a correlation between COVID-19 and TB, although with some bias in the results. Conversely, a population-based observational study reported decreased mortality rates among COVID-19 patients with concurrent TB.^[17]^ The inherent limitations of observational research may be the reason for these contradictory results, highlighting the need to establish causality between COVID-19 and TB. In this study, two-way causality was not supported by a thorough selection of IVs or different MR analysis techniques between COVID-19 and TB.

TB is an infectious bacterial disease caused by *Mycobacterium tuberculosis* that mainly affects the lungs and other parts of the body, including the central nervous system.^[18]^ However, the precise connection between the 2 remains unknown. Several factors may account for the relationship between COVID-19 and TB in observational studies. First, studies have confirmed a link between CD4 + TEM cell markers (GBP2 and LAG3) and the risk of pulmonary TB and COVID-19 infection,^[19]^ as the lungs are the primary sites affected by TB, and CD4 + T cells are closely related to TB. Khayat et al^[20]^ reported a case in which a patient suggestive of having TB experienced a drastic decrease in CD4 + T cells after COVID-19 infection, potentially leading to active TB. Second, a retrospective observational study by Karthik et al^[21]^ observed elevated levels of inflammatory markers, high neutrophil-to-lymphocyte ratio, and lymphopenia in 26 patients co-infected with COVID-19 and TB. Furthermore, compared to TB patients without COVID-19, co-infected patients had a longer disease course, increased treatment difficulty, and higher mortality rates. Studies have also suggested that transient immunosuppression associated with COVID-19 treatment may activate latent *M tuberculosis.*^[22,23]^

Further research into the interaction between COVID-19 and TB has shown that while both diseases can co-occur, the presence of COVID-19 does not necessarily exacerbate TB outcomes. A preliminary analysis of deaths in patients with both TB and COVID-19 indicated that the mortality rate was influenced more by age and the presence of comorbidities rather than a direct causal relationship between the 2 infections.^[24,25]^

This bidirectional MR analysis based on European population genetic data suggests that there may not be a two-way causality between COVID-19 and TB in this population. However, this study had some limitations. The limited availability of GWAS data for Asian populations restricts the generalizability of the findings because the analysis included people with only European ancestry. Future studies should use larger GWAS summary data and additional genetic tools for MR analyses to validate these results.

## Acknowledgments

We would like to thank Editage (www.editage.cn) for the English language editing. We would also like to thank Professor Bingxing Zhang for the statistical analysis.

## Author contributions

**Conceptualization:** Chidao Chen.

**Data curation:** Chidao Chen, Yanru Ji.

**Formal analysis:** Lei Wang, Fan Wang, Yanru Ji.

**Investigation:** Lei Wang, Chidao Chen, Fengzhu Sun, Yanru Ji.

**Methodology:** Chidao Chen, Peng Wang, Fengzhu Sun.

**Project administration:** Fan Wang, Zhonghao Huang.

**Resources:** Lei Wang.

**Software:** Peng Wang, Xixin Wang.

**Supervision:** Xixin Wang, Zhonghao Huang.

**Validation:** Peng Wang, Yanru Ji, Zhonghao Huang.

**Visualization:** Lei Wang, Peng Wang, Fan Wang.

**Writing – original draft:** Lei Wang.

**Writing – review & editing:** Zhonghao Huang.
